# Peri-operative fluid management in major colorectal surgery: do we comply with current guidance and what are the implications for our patients?

**DOI:** 10.1186/2197-425X-3-S1-A236

**Published:** 2015-10-01

**Authors:** L Vincent, H Vollmer, S McDouall

**Affiliations:** Oxford University Hospitals, Anaesthetics, Oxford, United Kingdom; Royal Berkshire Hospital, Reading, United Kingdom

## Intr

Individualised Goal Directed Fluid Therapy, monitoring Cardiac Output, or other surrogates of tissue oxygenation, to guide intra-venous (IV) fluid delivery, is a cornerstone of Enhanced Recovery. Debate surrounds the optimal timing, volume and targets for IV fluid, but the physiological consequences of head-down positioning, pneumoperitoneum (laparoscopic) and the implications of hypoperfusion or oedema for anastomotic integrity, demand scrupulous attention to fluid management in colorectal surgical patients. Flow monitoring devices (eg Oesophageal Doppler) can reduce complications and current guidance stipulates their use [1].

## Objectives

Our objectives were to review local peri-operative fluid management and assess the extent to which Goal Directed Fluid Therapy impacts on outcome in colorectal surgical patients.

- What is compliance with intra-operative Cardiac Output monitoring?

- How does Cardiac Output monitoring influence peri-operative fluid therapy and patient outcome?

## Methods

A retrospective notes analysis was performed on patients who underwent elective major colorectal surgery at the Royal Berkshire Hospital between January and May 2012. Demographics, co-morbidities and intra-operative and post-operative data were recorded, including haemodynamic monitoring, fluid management and post-operative destination. Outcomes recorded included post-operative complications, length of stay and 30-day mortality.

## Results

65 patients were included: mean age 67.6 years; 57% male.

5% operations were open, 77% laparascopic and 18% laparoscopic converted to open.

## Conclusions

- Cardiac Output monitoring is not used in a sufficient number of patients.

- Patients with Cardiac Output monitoring received more fluid intra-operatively, had higher complication rates and length of stay. This probably reflects a higher risk cohort, whose complexity is identified early and who are treated aggressively.

- Restriction of intra-operative IV fluid to 1 or 2 litres, made little difference to the total peri-operative intake.

- Post-operative fluid was better restricted in the High Dependency Unit.

To optimise fluid management through the peri-operative pathway, we propose:

- Intra-operative Cardiac Output monitoring should be universally implemented in major colorectal surgical patients.

- Patients should be nursed post-operatively in high dependency areas, which are equipped and staffed to facilitate tight, focused, individualised fluid management.

**Figure 1:**
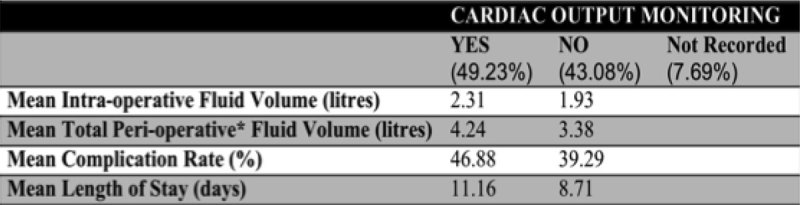
Use of Cardiac Output monitoring. For patients receiving < 1 or 1-2 litres intra-operative IV fluid, the mean total peri-operative* intake was 3.55 and 3.37 litres respectively. Patients receiving 2-3 or >3 litres intra-operatively received a mean total of 4.88 and 6.5 litres peri-operatively. * Intra-operative and 72 hours post-operative

**Figure 2:**
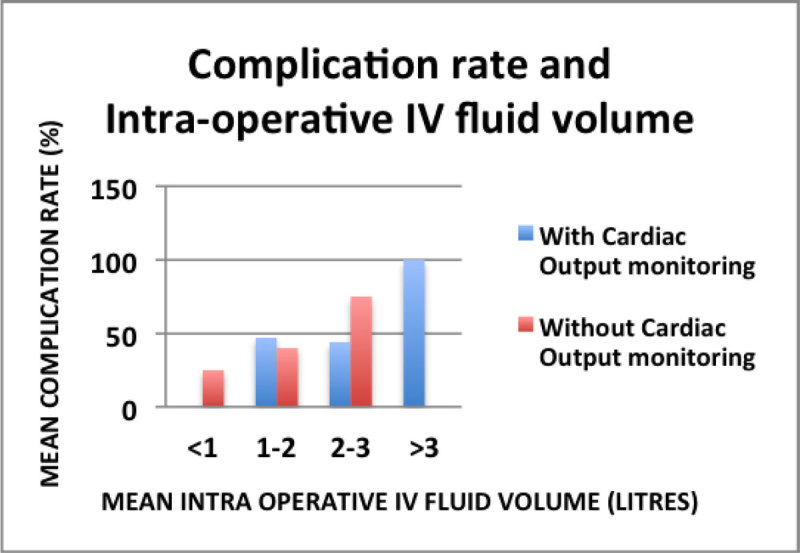
**Complication rate and Intra-operative IV fluid.**

**Figure 3:**
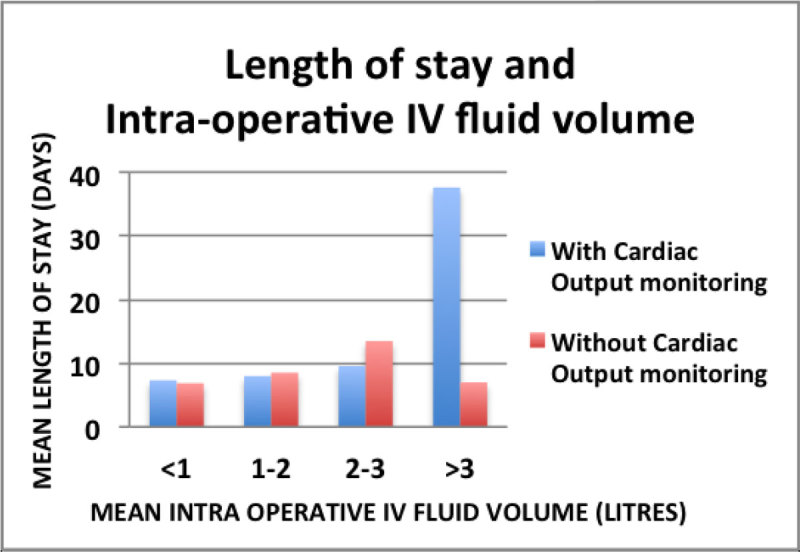
**Length of Stay and Intra-operative IV fluid. Patients admitted to High Dependency care received 0.89 litres IV fluid in the 72 hours post-operatively, compared to 2.19 litres when nursed in standard wards.**
